# The Predictive Role of Systemic Inflammatory Markers in the Development of Acute Kidney Failure and Mortality in Patients with Abdominal Trauma

**DOI:** 10.3390/jpm12122045

**Published:** 2022-12-10

**Authors:** Vlad Vunvulea, Ovidiu Aurelian Budișcă, Emil Marian Arbănași, Adrian Vasile Mureșan, Eliza Mihaela Arbănași, Klara Brînzaniuc, Raluca Niculescu, Iuliu Gabriel Cocuz, Adrian Dumitru Ivănescu, Ioana Hălmaciu, Lucian Mărginean, Réka Kaller, Eliza Russu, Bogdan Andrei Suciu

**Affiliations:** 1 Doctoral School of Medicine and Pharmacy, George Emil Palade University of Medicine, Pharmacy, Sciences and Technology of Targu Mures, 540142 Targu Mures, Romania; 2 Department of Radiology, Mures County Emergency Hospital, 540136 Targu Mures, Romania; 3 Department of Anatomy, George Emil Palade University of Medicine, Pharmacy, Science, and Technology of Targu Mures, 540139 Targu Mures, Romania; 4 Department of General Surgery, George Emil Palade University of Medicine, Pharmacy, Science, and Technology of Targu Mures, 540139 Targu Mures, Romania; 5 Department of Vascular Surgery, George Emil Palade University of Medicine, Pharmacy, Science, and Technology of Targu Mures, 540139 Targu Mures, Romania; 6 Clinic of Vascular Surgery, Mures County Emergency Hospital, 540136 Targu Mures, Romania; 7 Center for Advanced Medical and Pharmaceutical Research, George Emil Palade University of Medicine, Pharmacy, Sciences and Technology of Targu Mures, 540139 Targu Mures, Romania; 8 Faculty of Pharmacy, George Emil Palade University of Medicine, Pharmacy, Science, and Technology of Targu Mures, 540139 Targu Mures, Romania; 9 Department of Pathophysiology, George Emil Palade University of Medicine, Pharmacy, Science, and Technology of Targu Mures, 540139 Targu Mures, Romania

**Keywords:** abdominal trauma, acute kidney insufficiency, neutrophil-to-lymphocyte ratio, monocyte-to-lymphocyte ratio, platelet-to-lymphocyte ratio, systemic inflammatory index, systemic inflammatory response index, aggregate inflammatory systemic index

## Abstract

Background: Abdominal trauma is defined as a variety of injuries to the abdominal wall, solid or hollow intra-abdominal organs, and various intra-abdominal vessels. Recently, there has been a significant amount of interest in the establishment of a reliable biomarker that can predict the outcome in patients with an abdominal injury. The purpose of this study is to confirm the predictive role of inflammatory biomarkers and underlying risk factors and the risk of acute kidney insufficiency (AKI) developing and mortality in abdominal trauma patients; Materials and methods: The current study was intended as an observational, analytical, retrospective cohort study and included all patients over 18 years of age with a diagnosis of abdominal trauma confirmed through a CT scan admitted to the County Emergency Clinical Hospital of Targu-Mureș, Romania between January 2017, and December 2021; Results: Non-survivor patients had a greater age (*p* = 0.02), as well as a higher prevalence of ischemic heart disease (IHD) (*p* = 0.007), history of myocardial infarction (MI) (*p* = 0.002), peripheral arterial disease (PAD) (*p* = 0.01), chronic kidney disease (CKD) (*p* = 0.01), and all risk factors (*p* = 0.0004 and *p* < 0.0001). In terms of injured organs, we have in the second group a higher incidence of kidney injury (*p* = 0.003) and hemoperitoneum (*p* = 0.008). Multivariate analysis showed a high baseline value for all inflammatory biomarkers that are independent predictors of adverse outcomes for all recruited patients. Furthermore, for all hospitalized patients, the history of MI (*p* = 0.03; *p* = 0.001; and *p* = 0.003), PAD (*p* = 0.01; *p* = 0.01; and *p* = 0.002), obesity (for all *p* < 0.0001), CKD (*p* < 0.001; *p* = 0.01; and *p* = 0.001), and kidney injury (*p* = 0.02; *p* = 0.004; and *p* = 0.01) were independent predictors of all outcomes. Moreover, IHD (*p* = 0.008 and *p* = 0.02), tobacco (*p* < 0.0001 and *p* = 0.02), and hemoperitoneum (*p* = 0.009 and *p* = 0.01) were predictors of mortality and composite endpoint, but not for AKI risk, as well as atrial fibrillation [AF] (*p* = 0.04) as predictors of the composite endpoint Conclusions: Higher monocyte to lymphocyte ratio (MLR), platelets to lymphocyte ratio (PLR), systemic inflammatory index (SII), neutrophil to lymphocyte ratios (NLR), aggregate inflammatory systemic index (AISI), and systemic inflammatory response index (SIRI) levels at admission, according to our data, highly predict AKI risk and death.

## 1. Introduction

Abdominal trauma is defined as a variety of injuries to the abdominal wall, solid or hollow intra-abdominal organs, and various intra-abdominal vessels [1]. Depending on the mechanism of injury, abdominal trauma can be classified into blunt and penetrating trauma. Nevertheless, abdominal trauma is associated with high morbidity and mortality rates, the abdomen being the third most affected body region in trauma [2]. According to recent studies, abdominal trauma mortality rates are reported to range from 1 to 20% globally, which is largely due to population diversity [3,4,5,6,7,8]. Recent literature reports the spleen, liver, and kidney as being the most commonly affected organs [9,10,11].

We classify acute kidney insufficiency (AKI) as one of the most dangerous post-traumatic complications, with an incidence of up to 10% [12,13,14,15,16], and it can occur secondary to rhabdomyolysis in the case of crushing [17], or more commonly by impaired kidney perfusion [18].

Recently, there has been a significant amount of interest in the establishment of a reliable biomarker that can predict the outcome in patients with an abdominal injury. One of the most accessible biomarkers is the neutrophil-to-lymphocyte ratio (NLR). The ratio has been proven to be a valid predictor for the outcome of patients with COVID-19 infection [19,20,21,22], breast cancer [23], cardiovascular disease [24,25,26,27,28], and kidney disease [21,29]. Additionally, the platelet-to-lymphocyte ratio (PLR) is another widely researched biomarker, found to have great prediction power in the outcome of patients in the fields of oncology [30], orthopedy [25,31], and trauma care [32]. Moreover, the other hematological ratios, monocyte-to-lymphocyte ratio (MLR), systemic inflammatory index (SII), systemic inflammatory response index (SIRI), and aggregate inflammatory systemic index (AISI) have proved their prediction regarding the poor outcome in numerously chronic and acute pathologies [19,20,21,25,28]. The role of neutrophils, lymphocytes, and platelets in the modulation of inflammatory processes has been extensively described in the literature [33,34].

In works published by Bi et al. [35], Guangging et al. [36], Tang et al. [37], and Ntalouka et al. [38], the predictive role of NLR and PLR in the risk of AKI occurrence as an adverse event following gastrointestinal and hepatobiliary surgery, on-pump coronary artery bypass, and non-cardiac surgery patients, respectively, in the case of the endovascular treatment of aortic aneurysm, was demonstrated abdominally.

This study aims to verify the predictive role of inflammatory biomarkers and underlying risk factors and the risk of acute kidney insufficiency (AKI) developing and mortality in abdominal trauma patients.

## 2. Materials and Methods

### 2.1. Study Design

The current investigation was intended as an observational, analytical, and retrospective cohort study that included all patients over the age of 18 who had been diagnosed with abdominal trauma confirmed through a CT scan admitted to the County Emergency Clinical Hospital of Targu-Mureș, Romania between January 2017 and December 2021. Exclusion criteria were as follows: patients who died in the first 24 h, patients with bone fractures who required hospitalization in orthopedics, and patients with septic shock, hematological diseases, or thromboembolic events in the last two months.

Patients in the research were initially classified as “survivors” or “non-survivors” based on their bad prognosis during their hospitalization. To determine the risk of AKI, mortality, and a composite endpoint of AKI and mortality, the optimal cut-off values for NLR, MLR, PLR, SII, SIRI, and AISI were employed.

### 2.2. Data Collection

The patient’s age, sex, cardiovascular disease [atrial fibrillation (AF), arterial hypertension (AH), chronic heart failure (CHF), ischemic heart disease (IHD), history of myocardial infarction (MI), and peripheral arterial disease (PAD)], chronic kidney disease (CKD), diabetes mellitus (DM), chronic obstructive pulmonary disease (COPD), obesity (body mass index > 30), tobacco, and length of hospital stay (LOS) were extracted from the hospital’s electronic database. Moreover, the first blood test result extracted hemoglobin level, hematocrit, neutrophil, monocyte, lymphocyte, and platelet count, sodium, potassium, glomerular filtration rate (GFR), blood urea nitrogen (BUN), creatinine, and uric acid.

In terms of abdominal trauma at computed tomography (CT) scan, we recorded the presence of injury at the level of the following organs: liver, spleen, pancreas, kidney, small bowel, large bowel, and the presence of hemoperitoneum.

### 2.3. Systemic Inflammatory Markers

The first blood test result was used to determine the systemic inflammatory biomarkers, as follows:

− MLR = monocytes/lymphocytes
− NLR = neutrophils/lymphocytes
− PLR = platelets/lymphocytes
− SII = (neutrophils × platelets)/lymphocytes
− SIRI = (monocytes × platelets)/lymphocytes
− AISI = (neutrophils × monocytes × platelets)/lymphocytes

### 2.4. Study Outcomes

The primary endpoints were the risk of AKI, in-hospital mortality rate, and a composite endpoint of AKI and mortality. Outcomes were stratified for all optimal inflammatory biomarkers of cut-off values at baseline.

In terms of AKI classification, we used the kidney disease improving global outcomes (KDIGO) guidelines, based on the increased serum creatinine level or urine output range from stage I to III [39].

### 2.5. Statistical Analysis

SPSS for Mac OS version 28.0.1.0 was used for statistical analysis (SPSS, Inc., Chicago, IL, USA). Chi-square tests were used to assess the associations of all systemic inflammatory markers with category factors, while Student t-tests or Mann–Whitney tests were used to assess differences in continuous variables. To assess the predictive power and establish the cut-off of inflammatory markers, the receiver operating characteristic (ROC) curve analysis was utilized. The ROC curve analysis was used to determine the appropriate NLR, MLR, PLR, SII, SIRI, and AISI cut-off values based on the Youden index (Youden Index = Sensitivity + Specificity − 1, ranging from 0 to 1). A multivariate logistic regression analysis with variables with *p* < 0.1 was performed to find the independent predictors of the AKI risk, mortality, and a composite endpoint of the AKI and mortality.

## 3. Results

During the study period, 364 patients diagnosed with abdominal trauma met the inclusion criteria and followed up during hospitalization. The mean age was 42.83 ± 18.24 (18–89), and 258 patients were male (70.88%) (Table 1). During the hospitalization, 84 patients (23.07%) developed AKI, 81 patients died (22.25%), and 57 patients (15.65%) developed AKI and deceased later, respectively. In terms of AKI staging, 26 patients (7.14%) were stage I KDIGO, 31 patients (8.51%) were stage II, and 27 patients (7.41%) were stage III.

After we divided the patients according to survival status, we had a higher age in the non-survivor group (*p* = 0.02), as well as a higher incidence of IHD (*p* = 0.007), MI (*p* = 0.002), PAD (*p* = 0.01), CKD (*p* = 0.01), and all risk factors (*p* = 0.0004 and *p* < 0.0001). In terms of injured organs, we have in the second group a higher incidence of kidney injury (*p* = 0.003) and hemoperitoneum (*p* = 0.008). Moreover, severe variables from laboratory data were associated with poor outcomes: non-survivors had lower hemoglobin and hematocrit levels (*p* < 0.0001), lower GFR (*p* = 0.004) and lymphocyte levels (*p* < 0.0001), and higher neutrophils (*p* < 0.0001), monocyte (*p* < 0.0001), glucose (*p* = 0.02), PLT (*p* = 0.001), BUN (*p* < 0.0001), creatinine (*p* < 0.0001), uric acid (*p* = 0.02), and all hematological ratios (*p* < 0.0001). Additionally, the non-survivor patients had a higher incidence of AKI (*p* < 0.0001), composite endpoint (*p* < 0.0001), a long hospital stay (*p* < 0.0001), and stage II (*p* < 0.0001) and III (*p* < 0.0001) KDIGO. In contrast, there was a higher incidence of stage I KDIGO (*p* < 0.0001) in survivor patients. The rest of the comorbidities and laboratory data are presented in Table 1.

**Figure 1:**
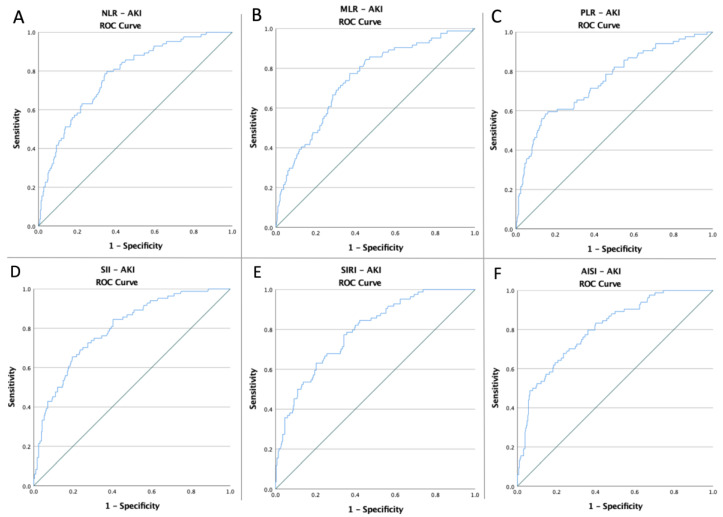
ROC curve analysis concerning the AKI risk (**A**) NLR (AUC: 0.777; *p* < 0.0001), (**B**) MLR (AUC: 0.744; *p* < 0.0001), (**C**) PLR (AUC: 0.751; *p* < 0.0001), (**D**) SII (AUC: 0.796; *p* < 0.0001), (**E**) SIRI (AUC: 0.790; *p* < 0.0001), and (**F**) AISI (AUC: 0.802; *p* < 0.0001); blue line – ROC curve; green line – diagonal line.

**Figure 2:**
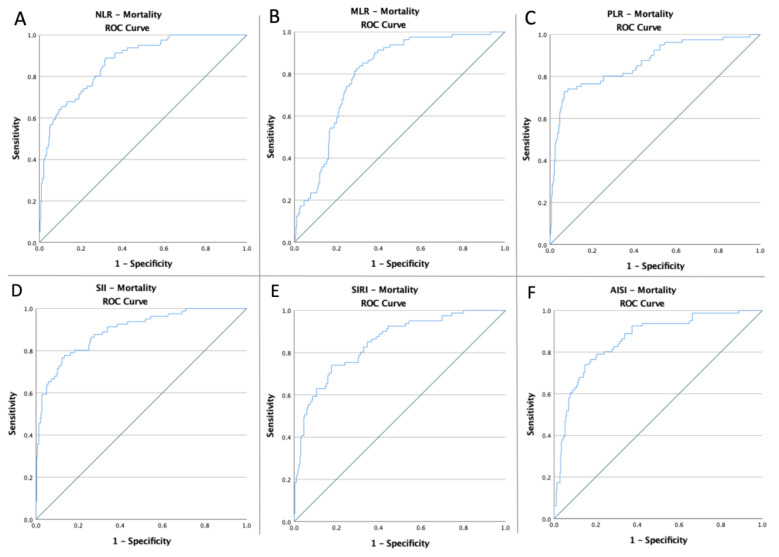
ROC curve analysis concerning the mortality (**A**) NLR (AUC: 0.870; *p* < 0.0001), (**B**) MLR (AUC: 0.800; *p* < 0.0001), (**C**) PLR (AUC: 0.865; *p* < 0.0001), (**D**) SII (AUC: 0.893; *p* < 0.0001), (**E**) SIRI (AUC: 0.846; *p* < 0.0001), and (**F**) AISI (AUC: 0.859; *p* < 0.0001); blue line – ROC curve; green line – diagonal line.

**Figure 3:**
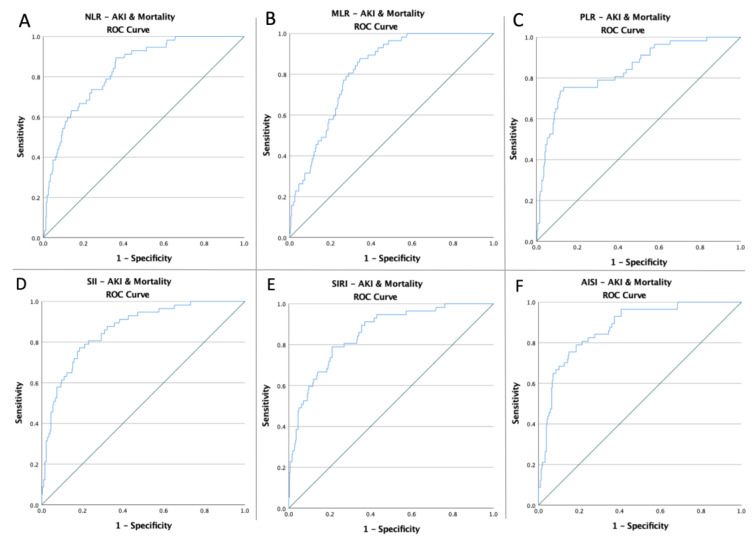
ROC curve analysis concerning the composite endpoint (**A**) NLR (AUC: 0.835; *p* < 0.0001), (**B**) MLR (AUC: 0.817; *p* < 0.0001), (**C**) PLR (AUC: 0.841; *p* < 0.0001), (**D**) SII (AUC: 0.862; *p* < 0.0001), (**E**) SIRI (AUC: 0.855; *p* < 0.0001), and (**F**) AISI (AUC: 0.873; *p* < 0.0001); blue line – ROC curve; green line – diagonal line.

To evaluate if the baseline of these indicators was predictive of AKI risk, death, and common endpoints in patients with abdominal injuries, receiver operating characteristic curves of all hematological ratios were generated (Figure 1, Figure 2 and Figure 3). Table 2 shows the ideal cut-off value determined by Youden’s index, the areas under the curve (AUC), and the prediction accuracy of the markers.

The results were subsequently evaluated after separating the patients into paired groups based on the optimal cut-off value of MLR, PLR, NLR, SII, AISI, and SIRI, according to the ROC. As seen in Table 3, there was a greater incidence of all poor outcomes for all inflammatory biomarkers studied.

A high baseline value for all of the studied markers was an independent prognostic factor of unfavorable outcomes for all enrolled patients, according to multivariate analysis (all *p* < 0.0001). Furthermore, for all hospitalized patients, the history of myocardial infarction (*p* = 0.03; *p* = 0.001; and *p* = 0.003), PAD (*p* = 0.01; *p* = 0.01; and *p* = 0.002), obesity (for all *p* < 0.0001), CKD (*p* < 0.001; *p* = 0.01; and *p* = 0.001), and kidney injury (*p* = 0.02; *p* = 0.004; and *p* = 0.01) were independent predictors of all outcomes. Moreover, IHD (*p* = 0.008 and *p* = 0.02), tobacco (*p* < 0.0001 and *p* = 0.02), and hemoperitoneum (*p* = 0.009 and *p* = 0.01) were predictors of mortality and composite endpoint, but not for AKI risk, as well as AF (*p* = 0.04) as predictors of the composite endpoint (Table 4).

## 4. Discussion

This research included 364 patients diagnosed with abdominal trauma. We identified the inflammatory biomarkers in all patients’ first admission blood test results and monitored the development of AKI, mortality rate, and a composite outcome of AKI and mortality. Our study’s most important outcome is that a high baseline value for NLR, MLR, PLR, AISI, SII, SIRI, cardiovascular disease (MI and PAD), and CKD are strong predictors of all outcomes. Additionally, the presence of a CT scan of kidney injury and obesity can predict all the outcomes. To the best of our knowledge, this is the first study to demonstrate that patients with high hematological ratios had a higher risk of AKI and intra-hospital mortality.

AKI is a well-known concern in polytraumatized patients, with an incidence of up to 36% [40,41,42,43,44,45], leading to a significant increase in mortality rate [46,47,48]. In the work published by Younan et al. [49], in which the dynamic evolution of NLR values in critically ill male trauma patients was analyzed, it was demonstrated that an increase in NLR in the first 48 h is associated with organ failure among male trauma patients. Moreover, Rau et al. [32], demonstrated that the low values of the total number of lymphocytes (OR: 1.1; *p* = 0.04) are a predictor of mortality in the case of 479 adult patients with polytrauma. Additionally, Ke et al. [50], demonstrated in univariate and multivariate analysis, that PLR (*p* < 0.001 and *p* = 0.02) is associated with an increased risk of mortality in the case of a group of 2854 adult trauma patients admitted to the intensive care unit.

According to the literature, the predictive values of hematological reports in polytraumatized patients have increasingly been studied, but with inconsistent findings. Additionally, the demand for prognostic tools in the negative evolution and decompensation of polytrauma patients has recently increased. High NLR values, according to Duchesne et al. [51], are related to early mortality in patients with severe post-traumatic hemorrhage who required a massive transfusion protocol. In contrast, Qiu et al. [52] discovered an association between NLR and the length of stay in the ICU and the duration of invasive mechanical ventilation, but not with mortality.

Regarding PLR, Li et al. [53] analyzed the predictive role of this marker in the case of 170 patients with traumatic brain injury, and in the multivariate analysis, they demonstrated that PLR (HR: 1.52; *p* = 0.009) is an independent predictor of short-term mortality.

Abu Alfeilat et al. [54], demonstrated that a value of NLR > 5.5, in the case of 294 patients who presented themselves to the emergency department, is a predictive factor in the case of the development of AKI (OR: 6.423; *p* = 0.031) in the multivariate analysis. Additionally, de Hond et al. [55], demonstrated that hematological ratios are associated with an increased risk of AKI and mortality in the case of a group of 1889 patients who presented to the emergency department with suspected infectious disease. Furthermore, Guangging et al. [36] observed high values of NLR in the group of patients who developed AKI after on-pump coronary artery bypass (2.63 vs. 2.06; *p* = 0.002).

In the current study, in the multivariate analysis, the high values of NLR (OR:7.09; *p* < 0.001 and OR:11.06; *p* < 0.001), MLR (OR:5.78; *p* < 0.001 and OR:11.14; *p* < 0.001), PLR (OR:5.89; *p* < 0.001 and OR:18.72; *p* < 0.001), SII (OR:6.76; *p* < 0.001 and OR:18.04; *p* < 0.001), SIRI (OR:6.25; *p* < 0.001 and OR:9.64; *p* < 0.001), and AISI (OR:6.08; *p* < 0.001 and OR:14.60; *p* < 0.001) are independent factors both for predicting the risk of developing AKI and for mortality during hospitalization. Moreover, the presence of PAD (OR:5.84; *p* = 0.01 and OR:6.14; *p* = 0.001), MI (OR:3.51; *p* = 0.03 and OR:7.64; *p* = 0.001), and obesity (OR:9.16; *p* < 0.001 and OR:9.67; *p* < 0.001) are associated with all recorded outcomes.

Although our findings are statistically significant, the study has a few limitations. Firstly, it is a retrospective and monocentric study with patient follow-up only throughout hospitalization. Future prospective multicenter studies with extended follow-ups are advised. Secondly, because the study was retrospective, we were unable to acquire information on chronic treatments used prior to admission (corticosteroids or anti-inflammatories meds). As a result, we were unable to determine how different drugs influence inflammatory biomarkers. Furthermore, there is no available information on the surgery, and we monitored the patients while they were in the hospital, but we are unsure of how many of them required chronic dialysis. In addition, more investigations are required to confirm our conclusions.

## 5. Conclusions

Higher levels of systemic inflammatory biomarkers upon admission, according to our data, greatly predict AKI risk and fatality. Additionally, myocardial infarction, obesity, renal damage, PAD, and CKD, were independent predictors of all outcomes in all hospitalized patients. Additionally, IHD, tobacco use, and hemoperitoneum have a predictive role in mortality and the composite endpoints, but not in AKI risk, while AF has a predictive role in the composite endpoint. Considering the simplicity of use and the low cost of these ratios, as well as the high risk of AKI development and mortality in trauma patients, they can be used to classify admission risk groups, improve patient treatment, and create predictive patterns.

## Figures and Tables

**Table 1:** Demographic information, comorbidities, risk factors, damaged organs, laboratory results, and outcomes were collected for all patients, and the two categories were separated based on poor outcomes.

Variables	All Patients *N* = 364	Survivors *N* = 283	Non-Survivors *N* = 81	*p* Value (OR; CI 95%)
Age mean ± SD (MIN–MAX)	42.83 ± 18.24 (18–89)	41.60 ± 17.61 (18–89)	45.80 ± 20.53 (20–88)	0.02
Male/Female SEX NO. (%)	258 (70.88%) 106 (29.12%)	198 (69.96%) 85 (30.04%)	60 (74.07%) 21 (25.93%)	0.47 (1.22; 0.70–2.14)
Comorbidities and Risk Factors				
AH, no. (%)	64 (17.58%)	47 (16.60%)	17 (20.98%)	0.36 (1.33; 0.71–2.7)
IHD, no. (%)	48 (13.18%)	30 (10.60%)	18 (22.22%)	0.007 (2.40; 1.26–4.59)
AF, no. (%)	14 (3.84%)	8 (2.82%)	6 (7.40%)	0.06 (2.75; 0.92–8.17)
CHF, no. (%)	24 (6.59%)	17 (6.007%)	7 (8.64%)	0.40 (148; 0.59–3.70)
MI, no. (%)	12 (3.29%)	4 (1.41%)	8 (9.87%)	0.002 (6.11; 1.94–19.24)
DM, no. (%)	38 (10.43%)	27 (9.54%)	11 (13.58%)	0.29 (1.48; 0.70–3.15)
COPD, no. (%)	10 (2.74%)	8 (2.82%)	2 (2.46%)	0.86 (0.87; 0.18–4.18)
PAD, no. (%)	8 (2.19%)	3 (1.06%)	5 (6.12%)	0.01 (6.14; 1.43–26.27)
CKD, no. (%)	20 (5.49%)	11 (3.88%)	9 (11.11%)	0.01 (2.55; 1.46–4.46)
Tobacco, no. (%)	16 (4.39%)	6 (2.12%)	10 (12.34%)	0.0004 (6.50; 2.28–18.49)
Obesity, no. (%)	17 (4.67%)	5 (1.76%)	12 (14.81%)	<0.0001 (9.66; 3.29–28.36)
Injured Organs				
Liver, no. (%)	130 (35.71%)	103 (36.39%)	27 (33.33%)	0.61 (0.87; 0.51–1.47)
Spleen, no. (%)	201 (55.21%)	160 (56.53%)	41 (50.61%)	0.34 (0.78; 0.48–1.29)
Pancreas, no. (%)	18 (4.94%)	14 (4.94%)	4 (4.93%)	0.99 (0.99; 0.31–3.12)
Large bowel, no. (%)	23 (6.31%)	17 (6.007%)	6 (7.40%)	0.64 (1.25; 0.47–3.28)
Small bowel, no. (%)	25 (6.86%)	20 (7.06%)	5 (6.17%)	0.77 (0.86; 0.31–2.38)
Kidney, no. (%)	23 (6.31%)	12 (4.24%)	11 (13.58%)	0.003 (3.54; 1.50–8.38)
Hemoperitoneum, no. (%)	191 (52.47%)	138 (48.76%)	53 (65.43%)	0.008 (1.98; 1.18–3.32)
Laboratory Data				
Hemoglobin g/dL median (Q1–Q3)	11.91 (10.47–13.36)	12.2 (10.5–13.55)	11.50 (10.4–12.7)	0.03
Hematocrit % median (Q1–Q3)	35.9 (31.4–39.98)	36.6 (31.36–40.66)	33.86 (31.6–37.2)	0.01
Glucose mg/dL median (Q1–Q3)	109 (93–140.75)	105 (92.5–132.9)	143 (104.25–170.5)	0.02
Sodium median (Q1–Q3)	138 (135–141)	138 (135–141)	138 (135–140.7)	0.35
Potassium median (Q1–Q3)	4.25 (3.74–5.0)	4.21 (3.72–5.19)	4.36 (3.9–4.74)	0.37
Uric acid median (Q1–Q3)	6.45 (5.1–8.2)	6.2 (5.0–7.95)	6.90 (5.5–8.6)	0.02
Bun mg/dL median (Q1–Q3)	67 (38.47–194.42)	56.5 (35.85–163.22)	145.5 (51–247.98)	<0.0001
Creatinine mg/dL median (Q1–Q3)	1.55 (0.86–6.1)	1.22 (0.84–5.68)	4.41 (1.36–9.57)	<0.0001
GFR (mL/min/1.73 m^2^) median (Q1–Q3)	74.17 (56.02–90.5)	76.72 (57.84–92.56)	66.52 (54.74–85.15)	0.004
Neutrophils ×10³/µL median (Q1–Q3)	8.24 (5.58–12.98)	7.43 (5.25–11.06)	12.94 (8.03–17.25)	<0.0001
Lymphocytes ×10³/µL median (Q1–Q3)	1.96 (1.47–2.66)	2.13 (1.68–2.88)	1.26 (0.98–1.89)	<0.0001
Monocyte ×10³/µL median (Q1–Q3)	0.9 (0.61–1.62)	0.8 (0.59–1.38)	1.29 (0.73–2.23)	<0.0001
PLT ×10³/µL median (Q1–Q3)	245.5 (200.6–303.85)	238.7 (193.15–300.4)	272 (222.7–316.1)	0.001
MLR, median (Q1–Q3)	4.15 (2.39–7.31)	3.36 (2.17–5.62)	9.79 (6.06–13.96)	<0.0001
NLR, median (Q1–Q3)	0.47 (0.30–0.94)	0.40 (0.28–0.68)	0.98 (0.67–1.80)	<0.0001
PLR, median (Q1–Q3)	120.03 (91.08–168.92)	110.66 (82.79–147.27)	224.05 (166.67–288.37)	<0.0001
SII, median (Q1–Q3)	1013.22 (583.19–1750.97)	757.46 (532.12–1392.7)	2725.15 (1697.4–3840)	<0.0001
SIRI, median (Q1–Q3)	4.96 (1.89–11.32)	3.44 (1.62–7.54)	15.56 (7.92–23.74)	<0.0001
AISI, median (Q1–Q3)	1163.85 (461.48–2874.06)	849.73 (377.96–1767.38)	3956.66 (2561.89–6192.3)	<0.0001
Outcomes				
AKI, no. (%)	84 (23.07%)	27 (9.54%)	57 (70.37%)	<0.0001 (5.57; 2.77–11.22)
AKI + Mortality, no. (%)	57 (15.65%)	0	57 (70.37%)	<0.0001 (5.57; 2.77–11.22)
Length of hospital stay, MEAN ± SD	9 (7–13)	9 (6–12)	10 (7–16)	<0.0001
Length of ICU stay, mean ± SD	7 (5–8.25)	6 (5–8)	8 (7–11)	<0.0001
AKI stage KDIGO				
0, no. (%)	280 (76.92%)	256 (90.45%)	24 (29.62%)	<0.0001
I, no. (%)	26 (7.14%)	18 (6.36%)	8 (9.87%)	0.26
II, no. (%)	31 (8.51%)	7 (2.47%)	24 (29.62%)	<0.0001
III, no. (%)	27 (7.41%)	2 (0.7%)	25 (30.86%)	<0.0001

AH = arterial hypertension; GFR = glomerular filtration rate; IHD = ischemic heart disease; AF = atrial fibrillation; BUN = blood urea nitrogen; CHF = chronic heart failure; MI = myocardial infarction; COPD = chronic obstructive pulmonary disease; PAD = peripheral arterial disease; CKD = chronic kidney disease; MLR = monocyte to lymphocyte ratio; PLR = platelets to lymphocyte ratio; NLR = neutrophil to lymphocyte ratio; AISI = aggregate index of systemic inflammation; SIRI = systemic inflammation response index; SII = systemic inflammatory index; AKI = acute kidney insufficiency; ICU = Intensive Care Unit; SD = standard deviation.

**Table 2:** ROC curves, ideal cut-off value, AUC, and prediction accuracy of inflammatory indicators in terms of outcomes.

Variables	Cut-off	AUC	Std. Error	95% CI	Sensitivity	Specificity	*p*-Value
AKI							
NLR	4.40	0.777	0.028	0.722–0.831	79.8%	64.6%	<0.0001
MLR	0.51	0.744	0.030	0.685–0.803	77.4%	62.9%	<0.0001
PLR	158.82	0.751	0.032	0.689–0.813	60.7%	78.9%	<0.0001
SII	1295.99	0.796	0.027	0.744–0.849	75.0%	69.3%	<0.0001
SIRI	5.57	0.790	0.027	0.738–0.843	78.6%	64.3%	<0.0001
AISI	1657.92	0.802	0.026	0.750–0.853	72.6%	69.6%	<0.0001
Mortality							
NLR	4.98	0.870	0.021	0.828–0.911	80.2%	73.1%	<0.0001
MLR	0.57	0.800	0.025	0.752–0.848	82.7%	70.3%	<0.0001
PLR	161.07	0.865	0.025	0.816–0.914	76.5%	85.2%	<0.0001
SII	1559.39	0.893	0.020	0.853–0.933	80.2%	81.6%	<0.0001
SIRI	7.85	0.846	0.024	0.798–0.894	75.3%	76%	<0.0001
AISI	2131.74	0.859	0.023	0.814–0.905	79%	79.5%	<0.0001
AKI and Mortality							
NLR	4.49	0.835	0.026	0.783–0.886	89.5%	63.8%	<0.0001
MLR	0.67	0.817	0.025	0.768–0.865	80.7%	71%	<0.0001
PLR	176.14	0.841	0.029	0.783–0.898	75.4%	86.6%	<0.0001
SII	1559.39	0.862	0.025	0.813–0.911	80.7%	76.9%	<0.0001
SIRI	10.08	0.855	0.026	0.804–0.906	78.9%	78.8%	<0.0001
AISI	2530.35	0.873	0.024	0.827–0.920	80.7%	78.5%	<0.0001

AISI = aggregate index of systemic inflammation; MLR = monocyte to lymphocyte ratio; PLR = platelets to lymphocyte ratio; NLR = neutrophil to lymphocyte ratio; SIRI = systemic inflammation response index; SII = systemic inflammatory index.

**Table 3:** Univariate analysis of all inflammatory biomarkers and adverse event occurrences in all patients over the study period.

	AKI	Mortality	AKI and Mortality
Low-NLR vs. high-NLR	17/197 (8.63%) vs. 67/167 (40.12%) *p* < 0.0001	16/223 (7.17%) vs. 65/141 (46.10%) *p* < 0.0001	6/202 (2.97%) vs. 51/162 (31.48%) *p* < 0.0001
Low-MLR vs. high-MLR	19/195 (9.74%) vs. 65/169 (38.46%) *p* < 0.0001	14/212 (6.60%) vs. 67/152 (44.08%) *p* < 0.0001	11/228 (4.82%) vs. 46/136 (33.82%) *p* < 0.0001
Low-PLR vs. high-PLR	33/254 (12.9%) vs. 51/110 (46.36%) *p* < 0.0001	19/260 (7.31%) vs. 62/104 (59.62%) *p* < 0.0001	14/280 (5.00%) vs. 43/84 (51.19%) *p* < 0.0001
Low-SII vs. high-SII	21/215 (9.77%) vs. 63/149 (42.28%) *p* < 0.0001	16/247 (6.48%) vs. 65/117 (55.56%) *p* < 0.0001	16/247 (6.48%) vs. 65/117 (55.56%) *p* < 0.0001
Low-SIRI vs. high-SIRI	19/200 (9.50%) vs. 65/164 (39.63%) *p* < 0.0001	20/235 (8.51%) vs. 61/129 (47.29%) *p* < 0.0001	12/254 (4.72%) vs. 45/110 (40.91%) *p* < 0.0001
Low-AISI vs. high-AISI	23/218 (10.5%) vs. 61/146 (41.78%) *p* < 0.0001	17/242 (7.02%) vs. 64/122 (52.46%) *p* < 0.0001	11/252 (4.37%) vs. 46/112 (41.07%) *p* < 0.0001

MLR = monocyte to lymphocyte ratio; PLR = platelets to lymphocyte ratio; NLR = neutrophil to lymphocyte ratio; AISI = aggregate index of systemic inflammation; SIRI = systemic inflammation response index; SII = systemic inflammatory index.

**Table 4:** Multivariate analysis of new adverse events occurred throughout the course of the research.

	AKI			Mortality			AKI AND Mortality		
	OR	95% CI	*p* Value	OR	95% CI	*p* Value	OR	95% CI	*p* Value
Age > 45	1.25	0.76–2.05	0.36	1.55	0.94–2.55	0.08	1.21	0.68–2.15	0.50
IHD	1.62	0.83–3.16	0.15	2.41	1.26–4.59	0.008	2.29	1.12–4.67	0.02
AF	2.61	0.88–7.76	0.08	2.75	0.92–8.17	0.06	3.18	1.02–9.87	0.04
MI	3.51	1.10–11.19	0.03	7.64	2.24–26.08	0.001	5.90	1.83–19.01	0.003
PAD	5.84	1.36–24.98	0.01	6.14	1.43–26.27	0.01	9.74	2.26–24.09	0.002
CKD	2.55	1.46–4.46	<0.001	3.09	1.23–7.74	0.01	3.21	1.97–6.50	0.001
Tobacco	2.73	0.98–7.58	0.053	6.50	2.28–18.49	<0.001	3.49	1.21–10.03	0.02
Obesity	9.16	3.12–26.86	<0.001	9.67	3.29–28.36	<0.001	9.11	3.30–25.12	<0.001
Kidney injury	2.77	1.17–5.68	0.02	3.54	1.50–8.38	0.004	3.17	1.28–7.89	0.01
Hemoperitoneum	1.27	0.78–2.08	0.32	1.98	1.19–3.32	0.009	2.20	1.21–4.03	0.01
high-NLR	7.09	3.94–12.74	<0.001	11.06	6.03–20.30	<0.001	15.09	6.24–36.09	<0.001
high-MLR	5.78	3.28–10.19	<0.001	11.14	5.94–20.92	<0.001	10.08	4.99–20.35	<0.001
high-PLR	5.89	3.42–9.77	<0.001	18.72	10.17–34.44	<0.001	19.92	10.02–39.60	<0.001
high-SII	6.76	3.88–11.79	<0.001	18.04	9.66–33.69	<0.001	13.90	6.83–28.25	<0.001
high-SIRI	6.25	3.54–11.02	<0.001	9.64	5.43–17.11	<0.001	13.96	6.98–27.92	<0.001
high-AISI	6.08	3.53–10.47	<0.001	14.60	7.95–25.81	<0.001	15.27	7.49–31.12	<0.001

AF = atrial fibrillation; IHD = ischemic heart disease; PAD = peripheral arterial disease; MI = myocardial infarction; CKD = chronic kidney disease; MLR = monocyte to lymphocyte ratio; PLR = platelets to lymphocyte ratio; NLR = neutrophil to lymphocyte ratio; AISI = aggregate index of systemic inflammation; SII = systemic inflammatory index; SIRI = systemic inflammation response index.

## Data Availability

Not applicable.
